# The ROOTS Parenting Intervention to Improve Child Emotional and Physical Health: Protocol for a Pilot Randomized Controlled Trial for Black and Latiné Families

**DOI:** 10.1111/jmft.70052

**Published:** 2025-07-04

**Authors:** Daniel K. Cooper, Francesca Lupini, Jayxa Alonzo, Michael Beets, Kate Guastaferro, Alexander C. McLain, Subina Saini, Ronald J. Prinz

**Affiliations:** ^1^ Department of Psychology and the Research Center for Child Well‐Being University of South Carolina Columbia South Carolina USA; ^2^ Department of Exercise Science University of South Carolina Columbia South Carolina USA; ^3^ Department of Social and Behavioral Sciences New York University New York New York USA; ^4^ Department of Epidemiology and Biostatistics University of South Carolina Columbia South Carolina USA

## Abstract

Black and Latiné children in the United States experience disproportionate rates of emotional and physical health problems, yet few preventive interventions address both types of outcomes in a culturally relevant way. This study explores the feasibility of a parenting program that incorporates ethnic‐racial socialization and healthy lifestyle behaviors to promote the health of Black and Latiné children ages 3–6. A type 1 hybrid randomized controlled trial will be conducted with 60 families, who will be assigned to a parenting program or an active control condition, assessed at three time points. Feasibility measures will include quantitative and qualitative assessments of implementation outcomes and determinants. The main intervention outcomes are child social‐emotional functioning, child healthy lifestyle behaviors, and parenting processes. Findings will inform future efforts to implement and scale culturally relevant parenting interventions aiming to improve the emotional and physical health of minoritized children.

**Trial Registration:**

The trial began recruiting in May 2024 (Trial Registry: NCT06111651, Version 5, June 13, 2024). Participants are expected to complete their 3‐month follow‐up assessments at the end of fall 2025.

## 1 Introduction

Minoritized children are at a heightened risk for various emotional problems (e.g., anxiety, depression, and psychological distress) and physical health issues (e.g., obesity) due to the known impact of individual and structural racism (Berry et al. 2021). Structural racism manifests in unequal access to high‐quality education, safe housing, healthy foods, and medical care, while individual‐level (or interpersonal) racism includes daily experiences of discrimination and bias—all of which have been linked to chronic stress, poor emotion regulation, and biological dysregulation in children (Williams et al. 2019). Black and Latiné children are disproportionately exposed to both forms of racism, which may help explain why they consistently experience the highest rates of many health problems (Berry et al. 2021). For example, Black and Latiné children have twice the rates of obesity as compared to their white counterparts From Crisis to Opportunity 2021). Black children between the ages of 3 and 17 also have 50% higher rates of behavior/conduct problems as compared to non‐Latiné White children (Bitsko et al. 2022). This mounting evidence has fueled recent calls to address health disparities by directly targeting the effects of racism in child health outcomes through culturally responsive interventions (Gordon 2020).

Family‐based preventive intervention is a promising strategy for preventing child health problems including social‐emotional problems (Menting et al. 2013; Sanders et al. 2014) and unhealthy lifestyle behaviors (e.g., sleep problems, sedentary behavior) (Soltero et al. 2021). However, preventive interventions focused on either or both of the aforementioned health domains have rarely included content to promote ethnic‐racial socialization, a potentially powerful protective factor for minoritized children. Ethnic‐racial socialization relates to how families teach their children about the values, traditions, and practices associated with their ethnic‐racial group and may play a key role in mitigating the negative effects of racism on health outcomes (Brody et al. 2021; Neblett et al. 2008) and in contributing to children's social‐emotional functioning (Umaña‐Taylor and Hill 2020) and healthy lifestyle behaviors (Conway et al. 2021; Granberg et al. 2009; Wang et al. 2021). The omission of ethnic‐racial socialization strategies from preventive parenting interventions is a crucial gap that may be limiting engagement, reach, and impact with minoritized families. Therefore, the main objective of the proposed project is to examine whether providing parenting support, with an added emphasis on ethnic‐racial socialization, improves social‐emotional functioning and healthy lifestyle behaviors in young Black and Latiné children.

### 1.1 Limitations of Existing Parenting Interventions for Supporting Child Health

Positive parenting practices, such as parental monitoring, positive reinforcement, and limit setting, are associated with greater child social‐emotional health (Leijten et al. 2019) and healthy lifestyle behaviors (Soltero et al. 2021). As such, many of the most effective child health‐promoting interventions teach positive parenting practices (Ling et al. 2017; Ochoa and Berge 2017; Smith et al. 2017). A key limitation of family‐based prevention efforts is that they have failed to incorporate ethnic‐racial socialization, a critical contributor to child health and well‐being. Two of the most effective ethnic‐racial socialization strategies parents can use are: (a) fostering racial‐ethnic pride and (b) preparing children to handle discrimination (Umaña‐Taylor and Hill 2020). However, these strategies have been largely left out of parenting interventions for minoritized groups. In fact, only 1 of the 36 evidence‐based family‐focused interventions listed on the Blueprints registry (Blueprints for Healthy Youth Development—Committed to Healthy Youth, Families and Communities n.d.) included content on ethnic‐racial socialization. This is a critical gap because positive ethnic‐racial socialization is linked to improved child psychosocial functioning and can protect against the harmful effects of racism (Brody et al. 2021). For example, a recent meta‐analysis found that increasing ethnic pride, one ethnic‐racial socialization strategy, was a robust predictor of positive child outcomes, including ethnic‐racial identity, self‐esteem, academic achievement, adaptive coping, and psychological well‐being (Umaña‐Taylor and Hill 2020). Although few studies have examined the link between ethnic‐racial socialization and child physical health, there is evidence that promoting ethnic‐racial socialization can buffer the negative effects of racism (Neblett et al. 2008, 2012). This is important because experiencing racism is shown to negatively impact physical health outcomes, such as increasing cortisol levels, body weight, and sleep problems (Cave et al. 2020). Therefore, incorporating ethnic‐racial socialization has the potential to enhance the efficacy of family‐based interventions for child health outcomes while trying to offset the effects of racism.

Another limitation in the field of family‐based prevention is that most studies have focused on addressing social‐emotional functioning and healthy lifestyle behaviors separately, rather than adopting a more holistic approach by integrating the two domains into a unified intervention. Addressing these two domains in separate interventions is likely more cumbersome for providers and participants and misses out on the potential interactive effects of targeting both domains in the same intervention. Multiple studies have found that parenting interventions aimed at decreasing disruptive behavior also had positive effects on child lifestyle behaviors, such as physical activity and sedentary behavior, highlighting the potential for cross‐over effects of parenting interventions (Brotman et al. 2012; Smith et al. 2015). Meta‐analytic findings suggest that interventions aimed at increasing children's healthy lifestyle behaviors (e.g., sleep, sedentary behavior, physical activity) were particularly effective when including parenting support (Ling et al. 2017). However, none of these studies involved a purposeful integration of evidence‐based practices to jointly support child physical and emotional health. Thus, there is a need for integrated family‐based interventions aimed at the dual promotion of child social‐emotional and physical health, as they may offer a more efficient, sustainable approach to prevention (Hurley et al. 2016).

Despite increasing interest in family‐based interventions to improve child health, there are two additional limitations in the literature that the current study seeks to address: most interventions have focused on (a) late childhood and adolescence (e.g., Migueles et al. 2019) or (b) youth who have already experienced problematic health outcomes (e.g., Menting et al. 2013). For example, four of the largest obesity prevention trials conducted in the United States, including COPTR (Pratt et al. 2013), GEMS (Obarzanek and Pratt 2003), TAAG (Webber et al. 2008), and Pathways (Caballero et al. 2003), focused on 7‐ to 13‐year olds. In terms of prevention scope, many of these efforts have utilized indicated prevention, which refers to programs designed specifically for individuals who already exhibit early signs of problems or elevated risk profiles (Muñoz et al. 1996). A recent review of parenting programs for reducing child disruptive behavior found that the vast majority involved indicated prevention, with only 8 of the 154 trials adopting a universal prevention perspective (Leijten et al. 2019). This imbalance presents a significant missed opportunity. Universal interventions are delivered to broad populations, regardless of individual risk, and are particularly well‐suited for public health promotion, as they reach children before symptoms emerge and can help shift population‐level outcomes (Matthay et al. 2021). Moreover, early childhood is a sensitive period for physical and psychosocial development (Dunn et al. 2019; Tierney and Nelson 2009). Implementing universal prevention efforts during this developmental window may lay a stronger foundation for long‐term health and well‐being and prevent future problematic trajectories.

### 1.2 Hybrid Effectiveness‐Implementation Studies

To continue to improve the delivery and reach of evidence‐based solutions for child health promotion, implementation science has emerged (Bauer et al. 2015). Implementation science seeks to accelerate the translation of evidence‐based practices into usual care (Bauer et al. 2015). This is particularly important in low‐resource settings, for which evidence‐based practices are often less feasible and effective (Chu and Weiser 2021). One promising strategy for accelerating the spread of evidence‐based practices is the type 1 hybrid effectiveness‐implementation approach, which simultaneously tests (a) intervention effectiveness and (b) its potential for widespread dissemination (Curran et al. 2022). This is often done by gathering implementation outcomes—such as providers' opinions on intervention acceptability, feasibility, and delivery cost—alongside the evaluation of intervention outcomes (Studts et al. 2022). Assessing implementation outcomes simultaneously can help create implementation strategies to address barriers to intervention delivery and improve the reach, impact, and sustainability of evidence‐based practices for minoritized children (Proctor et al. 2011).

### 1.3 Current Study Aims

The proposed study involves the development and preliminary evaluation of an integrated family‐based intervention (hereafter referred to as the ROOTS program) to promote the social‐emotional functioning and healthy lifestyle behaviors of Black and Latiné children ages 3–6. This hybrid effectiveness‐implementation study has the following aims: examine various implementation outcomes, such as the feasibility and acceptability of recruitment, retention, and fidelity, as well as potential barriers and facilitators to intervention delivery (Aim 1) and evaluate the pre‐post changes in child and parent outcomes as compared to an active control condition (Aim 2). If successful, this study can provide justification for larger efficacy trials that could ultimately lead to the availability of more holistic integrated interventions for promoting the health and well‐being of Black and Latiné children.

## 2 Methods

### 2.1 Study Setting and Design

Because the protocol will be implemented virtually, families living anywhere in the state of South Carolina will be able to participate. To pilot the feasibility of the ROOTS program, the study will use a longitudinal two‐arm randomized control trial design in which families are randomized into a parenting intervention (*n* = 30) or a control (*n* = 30) condition on a 1:1 ratio. We selected a nutrition control condition that is a similar length (6 weeks) and provides a similar structure (offers parents new strategies and a workbook to reflect on their progress and goals). This decision was based on empirical evidence suggesting a need for alternative approaches to intervention research to maximize participant engagement and minimize ethical concerns related to withholding services from underserved populations (Henry et al. 2017). Assessments will occur at three timepoints: pre‐intervention (T1), post‐intervention (T2), and 3‐month follow‐up (T3). After completing the third assessment, each condition will have the opportunity to receive the materials from the other condition.

**Figure 1:**
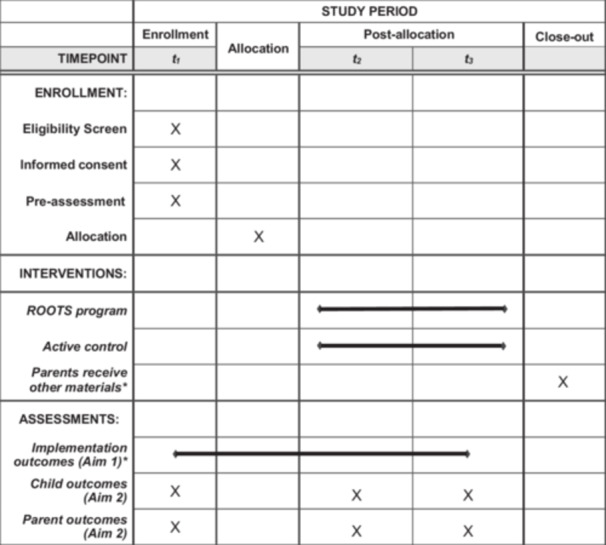
Schedule of enrollment, intervention conditions, and assessments. *After completing the third assessment, each condition will have the opportunity to receive the materials from the other condition. Implementation outcomes (e.g., eligibility, enrollment, and completion rates) will be collected on a continuous basis from enrollment to T3.

The hybrid effectiveness‐implementation approach adopted for this study examines intervention outcomes while concurrently assessing barriers and facilitators related to successful program delivery (see Figure 1). Blinding will occur at several levels, primarily with participants, outcome assessors, and data analysts. For example, intervention participants will not be aware of which intervention group is considered the treatment or control; rather, they will be instructed that the study seeks to compare the effects of two health interventions. Outcome assessment will be conducted using Qualtrics, which prevents potential bias from using unblinded assessors. Blinding will also occur at the data analyst level, specifically related to assessing outcomes.

### 2.2 Participants

Eligible parents must (a) live in South Carolina, (b) be able to speak English or Spanish, (c) have access to a device to be able to attend Zoom video calls, and (d) must not have received any parent training services or health services aimed at improving their children's behaviors or lifestyle habits, such as sleep or physical activity promotion programs, in the 12 months before enrolling in the study. Parents must also be the primary caregivers of a child that (e) is between 3 and 6 years of age, (f) is Black/African American or Latiné/Hispanic, and (g) their child must not have any psychological or chronic health issues that significantly interfere with their communication (e.g., child does not understand basic instructions from their parent) or that limit their ability to engage in physical activity (e.g., child uses a wheelchair). Primary caregiver refers to a parent who resides in the same household as the child for at least 50% of the time and has primary custody of the child. We chose to include children with physical or mental health diagnoses as long as these issues did not interfere with key intervention targets, such as their ability to benefit from parenting strategies and adjust their lifestyle behaviors (e.g., physical activity level). These criteria were selected because they align with our universal prevention approach that targets families across the risk spectrum. Target recruitment for this study is 60 families (*n* = 30 experimental, *n* = 30 control).

### 2.3 Recruitment and Randomization

Participants will be recruited using a multifaceted strategy. This involves distributing flyers via email to community partners and displaying them at various locations like schools, daycares, churches, grocery stores, and health clinics. We will also participate in community events, such as back‐to‐school gatherings. To further assist with recruitment, we will hire three designated recruiters who are well‐connected in the community and have experience as community health workers. These recruiters will also act as interventionists (also referred to as parent coaches) for our study. Interested parents can express their interest by calling our designated study phone number, sending an email, or completing an online eligibility form by scanning the QR code shown on the study flyer. After that, research assistants from the ROOTS team will confirm their eligibility during a screening call. Informed consent will be gathered through this screening call and by reviewing qualtrics online forms. Black and Latiné participants will be randomized separately to ensure adequate representation of each ethnic‐racial group within each treatment condition (participants of AfroLatiné descent will be randomized based on language preference). To create each randomization scheme, we will use Excel's random number generator function to produce and save 30 random numbers. These assignments will be hidden in Excel to prevent access by research staff making screening calls. After consenting to participate and completing the T1 assessment, participants will receive a study ID number. This ID number will be added to the appropriate randomization scheme (either Black or Latiné) in the order of enrollment. The random assignment will then be revealed by a designated study staff member.

### 2.4 Intervention Development and Delivery

#### 2.4.1 Program Development

To develop the ROOTS program, we utilized intervention mapping (Fernandez et al. 2019), a structured, theory‐ and evidence‐based approach for designing culturally responsive health promotion interventions. This process involved multiple steps, such as identifying target health outcomes (i.e., child emotional and physical health problems), determining malleable influences (e.g., parenting practices, ethnic‐racial socialization, and child health behaviors), selecting and integrating strategies, and planning for implementation and evaluation. For example, to gather information about child health and identify culturally relevant determinants, we conducted qualitative interviews with 47 community members, including Black and Latiné parents and community health workers (Cooper et al. 2024). Participants emphasized the need to integrate ethnic‐racial socialization, healthy lifestyle practices, and positive parenting into one coherent intervention. This community input directly shaped the content and delivery format of the ROOTS program.

#### 2.4.2 Program Content

ROOTS draws primarily from the Triple P—positive parenting program (Sanders 2012), an internationally disseminated intervention supported by over 200 randomized controlled trials and available in 24 languages (Sanders 2023). The ROOTS program integrates Triple P strategies with culturally grounded ethnic‐racial socialization content from one talk at a time (stein 2021; Soltero et al. 2021) and health promotion recommendations for young children (Allen et al. 2016; Brown et al. 2016; Jones et al. 2021; see Table 1). The six‐session curriculum teaches caregivers to use positive parenting techniques to support racial pride, prepare children for bias, and encourage healthy routines around sleep, screen use, and physical activity. The one talk at a time intervention was chosen for its feasibility with Black and Latiné families and for its compatibility with strategies taught in Triple P. While there are various cultural differences between non‐Latiné Black and Latiné families, there is evidence that these positive parenting and ethnic‐racial socialization strategies can be tailored to fit the needs of both groups. For example, strategies for promoting racial pride, such as celebrating cultural holidays, telling family stories, and reading books about culture, can apply to members of various racial or cultural groups, even though the cultural content within these strategies may differ (e.g., celebrating Juneteenth vs. Dia de los Muertos).

**Table 1:** Session‐by‐session outline.

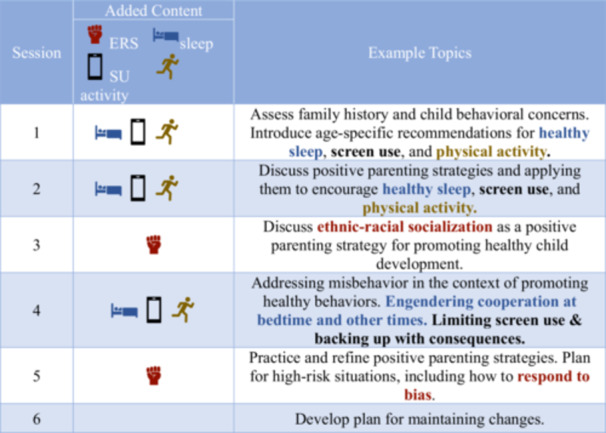

*Note:* Color‐coded emboldened text specifies the type of added content, such that red represents ethnic‐racial socialization content, blue represents sleep content, black represents screen use content, and gold represents physical activity content.

Abbreviations: Activity, physical activity; ERS, ethnic‐racial socialization; SU, screen use. [Color figure can be viewed at wileyonlinelibrary.com]

The ROOTS program's incorporation of healthy lifestyle behaviors is addressed mainly in Session 1, during which parents are educated on how sleep, screen time, and physical activity issues can contribute to child behavior problems. Parents learn about the current recommendations for promoting healthy child sleep habits (Allen et al. 2016), physical activity and sedentary behavior (Brown et al. 2016), and managing screen use (Jones et al. 2021). In later sessions, parents are taught positive parenting strategies that can be used to improve these lifestyle behaviors. For example, parents may be guided to use praise and behavioral reinforcement charts to encourage healthy sleep habits and to respond to bedtime misbehavior with consistent consequences such as planned ignoring or time out.

#### 2.4.3 Program Format and Delivery

ROOTS consists of 6 weekly one‐on‐one sessions, each lasting approximately 60–90 min. Sessions follow a structured format that includes a review of homework activities, didactic teaching, interactive discussions, and role play. Parents meet virtually via Zoom in either English or Spanish, depending on their preference. Sessions are led by trained interventionists who match the racial and ethnic backgrounds of participants. Parents receive a ROOTS toolkit by mail, which includes a workbook, culturally relevant children's books to support ethnic‐racial socialization, a program schedule, and study‐branded materials (e.g., handheld fan, hand sanitizer). Participants also receive weekly text message reminders about homework and appointment dates.

#### 2.4.4 Program Interventionists

Interventionists will be community‐integrated professionals (e.g., school counselors, community health workers) selected based on their shared cultural backgrounds with participants and prior experience working with families. These interventionists will also serve as designated recruiters for the study (described above). All interventionists will complete formal Triple P training (i.e., Standard Level 4 4‐day virtual training) and additional ROOTS‐specific training. This training will include: (a) reviewing the ROOTS facilitator manual, (b) viewing video demonstrations of each session led by the PI with a practice family, (c) participating in live role‐plays and supervision, (d) completing post‐session fidelity checklists where they self‐rate their coverage of topics, and (e) receiving ongoing consultation with the PI, consistent with the Triple P training model (Sanders et al. 2020).

#### 2.4.5 Nutrition Control Condition

Parents in the active control condition will receive a six‐session self‐directed nutrition intervention based on best practices for healthy eating. These materials were compiled from Myplate.gov and Nibbles for Health and cover a range of topics, including nutrition basics, trying new foods, breakfast and snacks, fruits and vegetables, protein and dairy, and bread and grains. Parents are given a workbook that includes empty spaces for goal setting and behavior tracking, as well as a study overview schedule and study swag. After the 3‐month follow‐up assessment (T3), parents in the nutrition control condition will receive the parenting intervention materials and have the option to attend a 1‐h consultation session to provide an overview of the materials.

### 2.5 Measures

#### 2.5.1 Feasibility and Implementation Outcomes

Two prominent implementation science frameworks will be used to guide the evaluation of feasibility and implementation outcomes: (a) the reach, effectiveness, adoption, implementation, maintenance (RE‐AIM; Glasgow et al. 1999) model to assess key implementation outcomes and (b) the consolidated framework for implementation research 2.0 (CFIR; Damschroder et al. 2022) to assess various determinants shown to impact intervention delivery. The RE‐AIM model is a prominent framework for assessing the relevance of interventions in practice settings and provides several important implementation outcomes to consider, such as evaluating whether the intervention was delivered as intended and measures related to feasibility, acceptability, adaptation, and fidelity. We will assess feasibility (or reach as defined in the RE‐AIM framework) using the following benchmarks (progression criteria) in accordance with the traffic light approach (Mellor et al. 2023): *feasibility of recruiting* (go/proceed with RCT: recruit ≥ 60 families in 12 months, amend/proceed with changes: recruit 40–50 families in 12 months, stop/do not proceed unless changes are possible: recruit ≤ 30 families in 12 months), *feasibility of enrollment* (go: ≥ 70% of eligible families enroll, amend: 40%–50%, stop: ≤ 30%), *feasibility of session completion* (go: ≥ 60% of randomized participants complete all six sessions, amend: 40%–50%, stop: ≤ 30%), and *feasibility of assessment completion* (go: ≥ 60% complete all three assessments, amend: 40%–50%, stop: ≤ 30%). These progression criteria were based on past family‐based intervention studies with minoritized samples (Smokowski et al. 2018; Weeland et al. 2025) and are intended to be interpreted as guidelines rather than strict rules. We recognize that some outcomes may fall between categories (e.g., 60% enrollment falls below the “go” threshold of 70% but above the “amend” threshold of 50%). In such cases, we will apply a structured consensus process involving multiple team members (e.g., PI, research staff, community partners) who review the full pattern of feasibility data across domains (e.g., enrollment, attendance, fidelity, and satisfaction). Decisions will be made based on the overall weight of evidence rather than any single metric (Mellor et al. 2023). For example, if enrollment falls into the “amend” range but other domains indicate strong feasibility (e.g., high session and assessment completion), the team may decide to proceed with minor adjustments rather than redesign the protocol. Conversely, consistent “amend” scores across domains may warrant amendments before proceeding. This approach allows for flexibility while ensuring that decisions remain transparent, data‐driven, and collaboratively reviewed.

Additionally, the CFIR framework provides 67 constructs presumed to impact intervention delivery, such as the characteristics of the intervention (or innovation), the delivery setting, and the recipient population, which have been shown to be particularly important considerations when working with underserved populations (Brownson et al. 2021). These CFIR constructs are assumed to be determinants of the implementation outcomes specified by the RE‐AIM model, helping to understand the conditions needed to support successful intervention delivery. Understanding which CFIR constructs influence adoption, implementation, and maintenance can inform the development of targeted strategies that balance scientific rigor with real‐world relevance. CFIR helps explain *why* implementation succeeds or fails, while RE‐AIM captures the practical outcomes of intervention delivery, such as the *who*, *what*, *where*, *how*, and *when* (King et al. 2020). Used together, these frameworks can strengthen implementation planning, improve fidelity and uptake, and support the long‐term sustainability of an intervention. In our study, we will use a combination of qualitative and quantitative measures to assess these various implementation outcomes (specified by RE‐AIM), as well as assess the intervention conditions that influence intervention success (specified by CFIR; see Table 2; King et al. 2020; Konrad et al. 2022).

**Table 2:** Implementation outcome table organized by RE‐AIM and CFIR domains.

Domain	Definition	Qual	Quant	Description of measures
RE‐AIM framework (implementation outcomes: who, what, where, how, when?)				
**R**each	Number, percentage, and representativeness of participants reached, including characteristics of nonparticipants		●	Record of recruitment, enrollment, and retention rates (for assessments and intervention) in accordance with our progression criteria
**E**ffectiveness	Impact of the intervention on outcomes of interest	●	●	*Quantitative*: T1, T2, and T3 measures of parent and child health outcomes (see Table 3)*Qualitative*: parent interviews assessing intervention satisfaction and perceived benefits
**A**doption	Number, percentage, and representativeness of settings and staff willing to adopt the intervention	●	●	*Quantitative*: interventionists’ satisfaction with the intervention (e.g., acceptability of intervention measure)*Qualitative*: in our formative work, we surveyed community health workers about the feasibility of ROOTS. We also plan to interview interventionists about their satisfaction with ROOTS and desire to implement it after the project ends
**I**mplementation	Degree to which the intervention is implemented as intended, including staff adherence to protocols, consistency of delivery, and associated time and cost	●	●	*Quantitative*: assessments of various aspects of fidelity (post‐session checklist, process quality measure)*Qualitative*: assess interventionists and participants on perceived barriers and facilitators for implementation
**M**aintenance	Degree to which the intervention is integrated into an organization's standard practices and long‐term operations	●	●	*Quantitative and qualitative*: assessments of interventionists’ willingness to continue implementing ROOTS and perceived ease of implementation (e.g., Feasibility of Implementation Measure)
CFIR framework (implementation conditions, barriers, facilitators)				
Innovation characteristics	Whether intervention demonstrates advantages over current practices and can be adapted for successful implementation in the target context	●		Qualitative interviews with parents and interventionists about perceived intervention barriers and facilitators, such as number and length of sessions, virtual format, ease of delivery
Outer setting	Whether implementing the intervention is important at this time, addresses a critical gap in care, and aligns with current priorities in the larger context (e.g., state)	●		Formative work involved assessing current community needs and delivery feasibility, including perspectives on parenting support, child health, and intervention characteristics and format (Cooper et al. 2024)
Inner setting	Whether implementing this intervention is feasible for a given setting (e.g., school, organization)	●		Formative work involved assessing community needs and delivery feasibility within various contexts. We will also interview interventionists about feasibility of ROOTS' virtual, community health worker delivery format.
Characteristics of individuals	Whether parents and providers view the intervention as valuable and relevant to families	●	●	Participants' and interventionists' perceptions of the acceptability and appropriateness of ROOTS for Black and Latiné families (acceptability of intervention measure; intervention appropriateness measure; interview questions on parents' engagement with ROOTS materials)
Implementation process	How implementing the intervention impacts current operating procedures and whose buy‐in, input, and expertise is needed to deliver and sustain the intervention	●	●	Qualitative and quantitative assessments with interventionists about perceived barriers and facilitators to intervention delivery, such as ease of implementation, costs, perception of community buy‐in (e.g., Feasibility of implementation measure)

*Note:* Qual = qualitative measure. Quant = quantitative measure. Some overlap exists between domains in the RE‐AIM and CFIR frameworks. For instance, RE‐AIM's *adoption* and *maintenance* dimensions align with CFIR's *inner setting* and *characteristics of individual* domains, as they reflect organizational readiness and individual perspectives. As a result, several measures correspond to more than one framework domain and are listed in multiple sections of the table to reflect this integration.

**Table 3:** Main and secondary outcome measures at T1, T2, and T3.

Construct	Measure	Reporter	Description
Main child outcomes			
Social‐emotional functioning	* *SDQ* (Goodman 1997)	Parent	25 items assessing emotional symptoms, peer problems, conduct problems, hyperactivity, and prosocial behaviors *α* = 0.88 (Palmieri and Smith 2007)
Sleep	Axivity	Objective measure	Average nightly sleep duration and quality over a 7‐day period (Martin and Hakim 2011)
	^**+**^ *PCSIS* (Alfano et al. 2013)	Parent	12 items assessing sleep‐related parenting behaviors and interactions, such as sleep dependence, sleep conflict, *α* = 0.82
Physical activity	Axivity	Objective measure	Accelerometer‐derived physical activity intensity (e.g., light, moderate) over a 7‐day period (Lynch et al. 2019)
Sedentary behavior/screen time	Axivity	Objective measure	Accelerometer‐derived sedentary time over a 7‐day period (Lynch et al. 2019)
Secondary parenting outcomes			
Ethnic‐racial socialization attitudes and practices	* *CSAB* (Derlan et al. 2016)	Parent	17 items assessing attitudes toward ethnic‐racial socialization (12 items) and their use of different ethnic‐racial socialization practices (five items) with their child, *α* = 0.86−0.92
	^+^ *RaSCS* (Anderson et al. 2020)	Parent	Adapted eight items assessing parents' confidence in using various ethnic‐racial socialization strategies
Parenting self‐efficacy	*PSAS (Dumka et al. 1996)	Parent	Five items assessing parents’ confidence in their parenting role, *α* = 0.79 (Matsuda et al. 2019)
Parenting practices	* *Parenting Scale* (Arnold et al. (1993))	Parent	30 items assessing various dimensions of maladaptive parenting discipline styles, such as laxness (permissive discipline) and over‐reactivity (anger, use of authoritarian discipline), *α* = 0.76−0.84 (López‐Fernández et al. 2022)

*Note:* ^+^Scale has been validated in English. *Scale has been validated in English and Spanish. Abbreviations: Axivity, axivity accelerometer; CSAB, cultural socialization attitudes and behaviors; PCSIS, parent–child sleep interactions scale; PSAS, parenting self‐agency scale; RaSCS, racial socialization competency scale; SDQ, strengths and difficulties questionnaire.

#### 2.5.2 Fidelity Monitoring

The plan for assessing fidelity is informed by Garbacz et al. (2014) and developed based on procedures associated with Triple P (e.g., Sanders et al. 2020), which includes measures of content fidelity (e.g., post‐session checklists, dosage, training providers) and process fidelity (e.g., process quality measure; Kirby and Sanders 2014). A post‐session checklist, similar to those used with Triple P and other evidence‐based interventions (Sanders et al. 2020), will be completed by the parent coaches after each session to determine whether the intended content was presented to participants. A separate team member will then review a randomly selected sample of recordings (25%) to provide a second assessment of content fidelity. Process fidelity will be assessed using the process quality measure and will also be completed for a random sample of sessions (25% of sessions).

#### 2.5.3 Intervention Outcomes

To assess preliminary intervention effects, this study will use a combination of validated parent‐report scales and objective measures to evaluate child and parent outcomes across three timepoints (T1–T3). Main child outcomes include social‐emotional functioning, sleep, physical activity, and sedentary behavior. Social‐emotional functioning will be measured using the strengths and difficulties questionnaire (Goodman 1997). Sleep, physical activity, and sedentary behavior will be measured using axivity accelerometers worn on the nondominant wrist for 7 consecutive days. Sleep variables (e.g., duration, quality) and physical activity variables (e.g., average minutes of sedentary, light, and moderate physical activity) will be derived using validated algorithms, such as the L512 algorithm within GGIR; Van Hees et al. 2015), and using age‐appropriate cut‐points (Migueles et al. 2019). We will assess the acceptability of the accelerometers by evaluating the number of full days (or nights) of wear data provided by each child. Secondary parenting outcomes will include parenting practices, ethnic‐racial socialization behaviors and attitudes, and parenting self‐efficacy. Each measure is described in Table 3, including psychometric properties and language validation status (English and/or Spanish). Measures not available in Spanish (i.e., parent–child sleep interactions scale, racial socialization competency scale) will be translated by bilingual study team members or a professional service using recommended procedures. This includes forward and back‐translation, reconciliation of discrepancies, and pilot testing to ensure cultural and linguistic appropriateness. This pilot study will also serve as an opportunity to examine the reliability and validity of these translated measures within our Spanish‐speaking sample.

#### 2.5.4 Demographics and Other Covariates

We will collect various demographic measures (at T1) and relevant covariates that have been shown to impact key study outcomes (T1–T3). Demographic measures will include characteristics such as parent and child gender, race/ethnicity, preferred language, years lived in the United States, age, and socioeconomic factors. Other covariates will include measures of parents' perceived discrimination (everyday discrimination scale; Williams et al. 1997), ethnic/racial identity (multidimensional inventory of black identity; Sellers et al. 1998), and childhood adversity exposure (expanded ACEs; Cronholm et al. 2015). These demographic and sociocultural factors have been consistently shown to be associated with parenting and child health (e.g., parent ACEs with harsh parenting; Wattanatchariya et al. 2024; parental discrimination and ethnic identity with ethnic‐racial socialization practices; White‐Johnson et al. 2010; socioeconomic status with child physical activity and behavioral problems; Gautam et al. 2023), and will therefore be examined as potential covariates or moderators in our analyses.

### 2.6 Data Collection

We plan to promote participant retention by offering an escalating compensation schedule (i.e., $75, $100, $125, for a total of $300) and encouraging participants to continue with assessments even if they drop out of the intervention. We will also send reminder emails or texts about session content, homework, and appointment dates. Each participant will be assigned a point person (a member of the research team) who will be available for questions via text throughout their time in the study. All survey assessments will be completed and stored using Qualtrics. To maintain confidentiality, only select members of the study team will have access to Qualtrics. The data will be cleaned and deidentified before completing statistical analyses.

### 2.7 Data Monitoring

Because of the low‐risk status of this pilot study, the data and safety monitoring plan for this trial focuses on close monitoring by the study principal investigator (PI), along with prompt reporting of excessive adverse events and any serious adverse events to the NIH and to the IRB at the University of South Carolina. The PI will monitor collective injury and other adverse event rates across all participants quarterly. If a larger than reasonably expected adverse event rate should occur—such as incidents of undue psychological distress, family conflict, or exacerbated child behavior problems resulting from intervention content as reported by parents—then the PI will comply with reporting requirements with respect to the IRB and NIH, as well as any potential actions regarding the stopping (or modifying) of the trial. Additionally, any protocol modifications will be communicated to the IRB and updated in the clinical trial registry.

### 2.8 Data Analysis Plan

To evaluate the feasibility of the ROOTS program (Aim 1), we will use a combination of descriptive statistics and qualitative methods. Descriptive analyses will report progression criteria, including recruitment, enrollment, attendance, and completion rates. We will also compute means, standard deviations, and response distributions on quantitative implementation measures, such as the acceptability of intervention measure (AIM), feasibility of intervention measure (FIM), and intervention appropriateness measure (IAM). To further assess implementation outcomes, we will analyze qualitative interviews from parents and interventionists using thematic analysis (e.g., Braun and Clarke 2006). These interviews will be independently coded by multiple members of the study team using the CFIR framework to identify salient barriers and facilitators across domains (e.g., intervention characteristics, inner and outer settings). We will also map emergent findings onto RE‐AIM dimensions (e.g., adoption, implementation, and maintenance) to assess feasibility and inform potential for scale‐up. Following independent coding, the team will engage in a structured consensus process to resolve discrepancies and refine themes. Final themes will be retained only if they (a) appear across multiple interviews and (b) are independently identified by all coders and agreed upon through consensus discussion.

Preliminary tests of ROOTS' effects (i.e., Aim 2) will be conducted using multilevel modeling (MLM; Fitzmaurice et al. 2011) to account for repeated measures nested within participants in R (R Core Team 2024). Separate mixed effects models will be estimated for each outcome: child social‐emotional functioning, child sleep, physical activity, and sedentary behavior, and parenting variables. Each model will include fixed effects for time (T1, T2, and T3), group (ROOTS vs. control), and the time × group interaction, with child age, gender, and various socioeconomic factors as covariates. All models will follow an intent‐to‐treat (ITT) design, including all randomized participants regardless of intervention completion (Hollis and Campbell 1999). Because this is a pilot study, emphasis will be placed on estimating effect sizes and 95% confidence intervals rather than formal hypothesis testing, consistent with best practices for early‐phase research (Thabane et al. 2010).

Accelerometer data will be downloaded and processed using the GGIR package in R (Van Hees et al. 2015). For sleep, we will use the L512 algorithm to estimate the primary sleep period and extract average nightly sleep duration and quality (e.g., wake after sleep onset). For physical activity, we will calculate daily time spent in sedentary, light, and moderate‐to‐vigorous intensity categories using preschool‐specific cut‐points validated for axivity devices (Migueles et al. 2019; Roscoe et al. 2017). Values will be averaged across all valid days at each timepoint. Finally, for descriptive purposes, we will conduct subgroup analyses based on delivery language (English vs. Spanish) and participant ethnic‐racial background (Latiné vs. non‐Latiné Black) by comparing the effect sizes of intervention outcomes across these groups. We will use restricted maximum likelihood (REML) estimation to analyze longitudinal outcomes, as REML provides less biased estimates of variance components than traditional maximum likelihood, particularly in models with small to moderate sample sizes and repeated measures. REML is well‐suited for linear mixed‐effects models because it accounts for the loss of degrees of freedom associated with estimating fixed effects, which improves the estimation of random effects and yields more reliable standard errors (McNeish 2017).

### 2.9 Dissemination Plans

The results of this study will be disseminated through various mediums and communicated to participants, community organizations, and the scholarly community. Proposed topics for presentation or publication will be shared with the PI, research lab, and the coordinating research center. For example, we will create infographics summarizing study results to send to participants and community organizations and post on social media. We plan to submit our findings to academic conferences and journal outlets. Data will be available on the Qualitative Data Repository, and study materials and full assessment protocols will be shared upon reasonable request to the first author. Statistical code used for the proposed analysis will be shared alongside the published findings.

## 3 Discussion

This study will provide needed information regarding whether ethnic‐racial socialization and healthy lifestyle behaviors can be integrated into a brief parenting intervention to enhance the health of Black and Latiné children. Specifically, the study will examine the acceptability, feasibility, and preliminary effects of the ROOTS program by tracking changes in longitudinal child and parent outcomes and gathering information about various implementation outcomes shown to influence intervention delivery, such as fidelity, acceptability, reach, and perceived barriers and facilitators. This proposal's integration of ethnic‐racial socialization—a key factor in buffering the negative effects of racism for minoritized groups—into a preventive parenting intervention represents one of the first efforts of its kind. This addition has the potential to enhance intervention engagement and bolster child health outcomes by mitigating the effects of racism on child health (Coard et al. 2021). For example, one study used data from two randomized clinical trials of a parenting intervention that incorporated ethnic‐racial socialization and found that the intervention buffered the effects of prior discrimination on various child outcomes (Brody et al. 2021).

Many family‐based health promotion interventions have focused on the emotional and physical health domains separately, rather than in the same intervention. A low‐dose, early, preventive intervention offers a solution to several of the limitations of past family‐based interventions. Because parenting practices can be used to promote a wide range of child health behaviors, an integrated parent training intervention has the potential to impact children's physical and emotional health while minimizing the added burden on parents and providers.

One potential concern is that the dose of the parenting intervention would be insufficient to produce the expected changes in certain target outcomes (i.e., ethnic‐racial socialization and healthy lifestyle behaviors). This is particularly relevant for the healthy lifestyle content, as other interventions for this domain are often more intensive. However, this concern might be tempered by the inclusion of well‐established determinants of child health outcomes (e.g., positive parenting, ethnic‐racial socialization), which could have synergistic effects on study outcomes, thereby bolstering program effects. The proposed intervention also differs from past family‐based interventions, in that it is focused on universal prevention and, therefore, requires a less intensive intervention as compared to other prevention approaches (e.g., indicated prevention) that solely target individuals experiencing higher levels of risk.

### 3.1 Implications and Conclusion

This intervention protocol can serve as a valuable resource for program evaluators, intervention developers, and clinical researchers interested in implementing culturally responsive strategies for family‐based health promotion. We provided important behind‐the‐scenes details about the processes and decisions often involved in complex intervention development, which are frequently left out of existing literature. For example, numerous scholars have highlighted the lack of transparency in intervention development, calling it the “Cinderella” black box (Murray et al. 2023). This makes it challenging for interested parties to learn from or replicate intervention development strategies. Importantly, our protocol outlined the various steps taken to develop the ROOTS program, guided by an intervention mapping approach, such as identifying key determinants of child health and selecting evidence‐based strategies. We also considered the unique needs of Black and Latiné families and selected positive parenting and ethnic‐racial socialization strategies that could be tailored to both groups. Such structured, culturally attuned approaches to intervention development can help improve the quality and reach of family‐based interventions for minoritized populations. Overall, the expected contribution of our project is to provide important foundational knowledge regarding the feasibility of combining ethnic‐racial socialization and healthy lifestyle behaviors into a brief parenting intervention to enhance minoritized children's physical and emotional health. If successful, the results of this pilot trial could lead to advancements in prevention efforts focused on improving child health‐related outcomes, and ultimately lead to decreased health inequities for minoritized children.

## Ethics Statement

The study protocol was approved by the University of South Carolina's Institutional Review Board (IRB Protocol #Pro00124236) and aligns with the SPIRIT reporting guidelines (Chan et al. 2013). All participants will provide consent to participate in the study. Consent for publication will not be required because no identifying information will be included.

## Data Availability

Data will be made available in an online repository and other materials can be shared upon reasonable request.
